# The Effect of Timing of Tetanus-Diphtheria-Acellular Pertussis Vaccine Administration in Pregnancy on the Avidity of Pertussis Antibodies

**DOI:** 10.3389/fimmu.2019.02423

**Published:** 2019-10-11

**Authors:** Bahaa Abu-Raya, Michelle L. Giles, Tobias R. Kollmann, Manish Sadarangani

**Affiliations:** ^1^Vaccine Evaluation Center, BC Children's Hospital Research Institute, Vancouver, BC, Canada; ^2^Division of Infectious Diseases, Department of Pediatrics, University of British Columbia, Vancouver, BC, Canada; ^3^Department of Obstetrics and Gynaecology, Monash University, Melbourne, VIC, Australia

**Keywords:** immunization, pertussis, gestation, avidity, pregnancy

## Abstract

**Background:** Optimal timing of gestational tetanus-diphtheria-acellular pertussis (Tdap) vaccination is not well-defined. No well-established specific anti-pertussis antibody level correlates with protection, suggesting the importance of antibody quality such as avidity. We aimed to determine the effect of timing of vaccination with Tdap in pregnancy on the avidity of cord anti-pertussis toxin (PT) immunoglobulin G (IgG).

**Methods:** Prospective study of newborns in a tertiary hospital (Melbourne, Australia) born to women vaccinated with Tdap in pregnancy. Ammonium thiocyanate was used as a bond-breaking agent to measure the avidity of anti-PT IgG using concentrations between 0.25 M (to measure low avidity antibodies) and 3 M (to measure very high avidity antibodies). Anti-PT IgG levels achieved at each ammonium thiocyanate concentration in cord samples of women vaccinated during 28–32 weeks gestation (WG) vs. 33–36 WG, and women vaccinated 5–12 vs. 1–4 weeks prior to delivery were compared using *t*-tests.

**Results:** Newborns of women vaccinated with Tdap during 28–32 WG (*n* = 43) had statistically significant higher concentrations of medium and high avidity anti-PT IgG compared with newborns of women vaccinated during 33–36 WG (*n* = 47), 11.6 IU/ml (95% CI, 8.8–15.2) IU/ml vs. 6.7 IU/ml (95% CI, 5.2–8.6) and 10.1 IU/ml (95% CI, 7.4–13.8) vs. 5.7 (95% CI, 3.6–8.9) IU/ml (*p* = 0.007 and *p* = 0.035), respectively. Newborns of women vaccinated 5–12 weeks before delivery (*n* = 64) had statistically significant higher concentrations of high and very high avidity anti-PT IgG compared with newborns of women vaccinated within 4 weeks before delivery (*n* = 25), 10.3 IU/mL (95% CI, 7.9–13.4) vs. 3.3 IU/mL (95% CI, 1.7–6.4), 12.6 IU/mL (95% CI, 9.4–16.9) vs. 4.3 IU/mL (95% CI, 2.2–8.5) (all *p* < 0.03), respectively.

**Conclusions:** Quantification of levels of anti-PT IgG with different avidities demonstrated that pertussis vaccination 5–12 weeks before delivery was associated with higher anti-PT IgG avidity compared with vaccination within 4 weeks before delivery. Pertussis vaccination during 28–32 WG was associated with higher anti-PT IgG avidity compared with vaccination during 33–36 WG, supporting vaccination at 28–32 over 33–36 WG for optimal protection against pertussis in infancy.

## Introduction

Infants under 3 months of age suffer substantial morbidity and mortality from pertussis disease (1, 2). Vaccination of all pregnant women with tetanus-diphtheria-acellular pertussis (Tdap) vaccine is recommended in an increasing number of countries, including the UK (3), USA (4), Australia (5), Canada (6), Brazil (7), and Argentina (8) to protect infants too young to be vaccinated themselves. The optimal timing of vaccination in pregnancy to provide maximal protection to young infants remains an important knowledge gap (9) leading to variable national recommendations, for example 16–32 weeks gestation (WG) in the UK and 27–32 WG in Canada (3–6). In addition, although vaccination against pertussis in pregnancy has been shown to be effective in preventing pertussis disease in infancy, breakthrough cases do occur in infants born to women vaccinated according to their national recommendations (10–15), suggesting the need for data to further understand the variables effecting vaccine effectiveness. The impact of timing of Tdap vaccination during pregnancy on the infants' immunity to pertussis is one of the important variables.

Higher anti-pertussis antibody levels are associated with clinical protection from pertussis disease (16), but there is no specific anti-pertussis antibody level that correlates with protection. This emphasizes the importance of evaluating anti-pertussis antibody function and not only antibody concentration. Antibody avidity is one important measure of antibody quality, which examines the overall binding strength between a specific antibody and a target antigen (17, 18). Antibody functions have been established as a correlate of post-vaccination protection from other bacterial invasive diseases (e.g., bactericidal antibody in meningococcal disease) (19) and are likely also be important for protection against pertussis disease.

Previous studies evaluating the avidity of vaccine-induced antibodies are based on a comparison of antibody levels with vs. without the addition of a single concentration of bond-breaking agent, leading to an arbitrary and artificial separation of antibodies into “low” and “high” avidity antibodies (17, 18, 20). However, immune response to vaccination is polyclonal and results in antibodies with different avidities. No published studies have assessed the full avidity profile of anti-pertussis antibodies after vaccination in general and in pregnancy in particular (21–23). Assessing the full spectrum of antibody avidity after vaccination will provide comprehensive insight on avidity maturation after vaccination.

Data on the effect of timing of antenatal pertussis vaccination on the avidity of anti-pertussis antibodies are scarce, conflicting and derived from two small studies which assessed avidity with limited approach of using a single concentration of bond-breaking agent (24, 25). Acellular pertussis vaccines contain components of *B. pertussis* such as pertussis toxin (PT), filamentous haemagglutinin, fimbrial antigens, and pertactin. PT is an important virulence factor of *B. pertussis* (26, 27). PT is thought to be the cause of leukocytosis (28–31), which is associated with poor outcome among infants with pertussis (32, 33). Anti-PT antibodies are thus important in protecting from pertussis disease.

In this study, we aimed to determine the effect of timing of vaccination with Tdap in pregnancy on anti-PT antibodies conveyed to the newborn at the time of delivery in cord blood of a large cohort of pregnant women, evaluating and contrasting both antibody concentration as well as avidity.

## Methods

### Study Design

Pregnant women at a tertiary obstetric hospital (Monash Health, Melbourne, Australia) were prospectively recruited (April–September 2014) as previously reported (34). Inclusion criteria were healthy pregnant women with a singleton pregnancy between 28 and 36^+6^ WG (34). Women were excluded if they had one or more of the following: receipt of Tdap vaccine during their current pregnancy; immunosuppressive disorder; high risk for preterm delivery.

Women who declined to receive Tdap but were willing to participate in the study were the unvaccinated control group.

### Laboratory Analysis

Serum was separated from cord blood by centrifugation at the time of collection and stored at −80°C. Samples were shipped in temperature-controlled conditions to the Vaccine Evaluation Center (Vancouver, Canada) for avidity analysis.

### Determination of Anti-pertussis Toxin (PT) IgG Antibody Avidity

Avidity analysis of anti-PT IgG was performed as previously described using anti-PT IgG ELISA (EUROIMMUN) with ammonium thiocyanate (NH_4_SCN) (SIGMA–ALDRICH, St. Louis, MO) as a bond-breaking agent (21, 24). A range of ammonium thiocyanate concentrations was used [0.25 molar (M), 0.5, 1, 1.5, 2, 3 M]. All standards, controls and study samples were analyzed in duplicate with the average of the two samples taken as the final value.

### Calculation of Relative Avidity Index (RAI), Fractional and Total RAI of Anti-PT IgG

The Relative Avidity Index (RAI) for every sample at each ammonium thiocyanate concentration was calculated and expressed as a percentage (Supplementary Tables 1a,b). Samples not treated with ammonium thiocyanate or treated with the lowest ammonium thiocyanate concentration (0.25 M) with optical density values lower than the ELISA's lower levels of quantification were excluded from further avidity analysis.

The fractional RAI of anti-PT IgG achieved at a specific ammonium thiocyanate concentration was calculated (Supplementary Tables 1a,b). Within the range of concentrations of ammonium thiocyanate used (0.25–3 M), our data demonstrated high linear correlation between increasing ammonium thiocyanate concentration and decreasing RAI (*r* = −0.88, *p* < 0.001). Thus, a total RAI value of anti-PT IgG that reflected the weighted contribution of the fractional RAIs of anti-PT IgGs achieved at the specific ammonium thiocyanate concentrations was calculated (Supplementary Tables 1a,b) and expressed in Avidity Units (AU).

### Quantification of Fractional and Total Absolute Avidity Levels of Anti-PT IgG

The fractional absolute avidity levels of anti-PT IgG achieved at a specific ammonium thiocyanate concentration was quantified and expressed in IU/mL (Supplementary Tables 1a,b). The quantified fractional absolute avidity levels of anti-PT IgG at 0.25, 0.5, 1, 1.5, 2, and 3 M of ammonium thiocyanate were classified as low, low–medium, medium, medium–high, high, and very high avidity anti-PT IgG antibodies, respectively. The levels of anti-PT IgG eluted by the lowest ammonium thiocyanate concentration (0.25 M) were classified as very low avidity anti-PT IgG antibodies. The total absolute avidity levels of anti-PT IgG reflecting the weighted contribution of the fractional absolute avidity levels of anti-PT IgG were calculated and expressed in Absolute Avidity Units (AAU)/mL (Supplementary Tables 1a,b). Anti-PT IgG levels measured without the addition of ammonium thiocyanate (T_0_ in Supplementary Tables 1a,b), were referred to as total anti-PT IgG.

### Statistical Analyses

The demographic and baseline characteristics of the entire cohort have been published (34). In this study, we report only data for participants included in the avidity analysis. The demographic and baseline characteristics of pregnant women vaccinated during 28–32 WG, 33–36 WG (per the original study classification) (34) and unvaccinated women and their newborns were compared using Pearson's chi-squared test for categorical variables, and one-way analysis of variance for normally distributed continuous variables. The demographic and baseline characteristics of pregnant women vaccinated during 28–32 WG and 33–36 WG and their newborns were compared using Pearson's chi-squared test for categorical variables, and independent sample *t*-test for normally distributed continuous variables. The natural log of total anti-PT IgG levels, fractional absolute avidity levels of anti-PT IgG and total absolute avidity levels of anti-PT IgG were used for further analysis.

Anti-PT IgG levels and the total RAI of anti-PT IgG were compared between newborns of women vaccinated with Tdap vs. unvaccinated women, and newborns of women vaccinated during 28–32 WG vs. 33–36 WG using independent sample *t*-tests. In addition, newborns were classified according to time elapsed between maternal Tdap vaccination in pregnancy and delivery into two groups: 1–4 and 5–12 weeks prior to delivery. Anti-PT IgG levels and the total RAI of anti-PT IgG were compared between newborns of women vaccinated 1–4 vs. 5–12 weeks prior to delivery using independent sample *t*-tests. Univariate linear regression analysis was used to identify baseline characteristics variables that could potentially impact anti-PT IgG levels and total RAI of anti-PT IgG. This was followed by multivariable linear regression models in which the response variable was anti-PT IgG levels or total RAI of anti-PT IgG levels, and all variables identified in the univariate regression model with *p* ≤ 0.25 were included. In all models, the variable of interest [Tdap vaccination status in pregnancy (Tdap vaccinated or Tdap unvaccinated) or timing of vaccination in pregnancy] was also included. Pearson correlation assessed the relationship between the timing of Tdap vaccination in pregnancy (in WG) or the time interval between vaccination and delivery (in weeks) and anti-PT IgG levels as well the total RAI of anti-PT IgG. Density estimates of total absolute avidity levels of anti-PT IgG according to timing of vaccination in pregnancy were performed using Gaussian kernels.

Unsupervised clustering of the fractional absolute avidity levels of anti-PT IgG for all newborns were performed. The timing of Tdap administration in pregnancy and time interval between vaccination and delivery were independently displayed to enable visualization of the relationship between clinical variables and clusters of anti-PT IgG avidity profiles. R version 3.4.0 was used for all analyses.

### Ethical Aspects

The original study was approved by Monash Health Human Research Ethics Committee (HREC Ref: 13426B) and all participants provided informed and signed consent (34). The current study was approved by University of British Columbia Children's and Women Research Ethics Board (Certificate number: H17–00050).

## Results

In the original study, where only samples with paired maternal–cord sera were analyzed for total anti-PT IgG levels, analysis was performed on 29 samples for unvaccinated women, 42 samples for women vaccinated during 28–32 WG and 45 for women vaccinated during 33–36 WG. In this study, a total of 125 cord serum samples were available for avidity analysis (33 for unvaccinated women, 44 vaccinated with Tdap between 28 and 32 WG and 48 vaccinated with Tdap between 33–36 WG) and 112 were included in the final analysis (Supplementary Figure 1). In this subgroup of women, there were no significant differences in the baseline characteristics of pregnant women vaccinated during 28–32 WG compared with pregnant women vaccinated during 33–36 WG (Supplementary Table 2).

### Anti-PT IgG Avidity by Vaccination Status in Pregnancy

Newborns of women vaccinated with Tdap in pregnancy had higher total anti-PT IgG levels, total RAI of anti-PT IgG, total absolute avidity levels of anti-PT IgG, and fractional absolute levels of low–medium, medium, medium–high, high, and very high avidity anti-PT IgG compared with newborns of unvaccinated women, after adjustment to multiple variables (Table 1). To investigate the potential effect of pre-pregnancy pertussis vaccination status on anti-PT IgG levels at delivery, we compared total anti-PT IgG levels in women vaccinated against pertussis during 5 years before pregnancy to levels of anti-PT IgG in women not vaccinated against pertussis in the past, vaccinated more than 5 years before pregnancy or their vaccination status was not determined (per Supplementary Table 2). The GMC of total anti-PT IgG was 25.4 IU/mL (95% CI: 17.3–37.2) vs. 18.6 IU/mL (13.4–25.9), in the former vs. the latter group, respectively, *P* = 0.243.

**Table 1:** Cord anti-PT IgG levels with different avidities of women vaccinated with Tdap during pregnancy and unvaccinated women.

	**Vaccinated during pregnancy (*n* = 90)**	**Unvaccinated during pregnancy (*n* = 22)**	***P***	Adjusted P^*^
Total anti-PT IgG levels (IU/mL), GMC (95% CI)	62.5 (52.3–74.8)	21.4 (16.6–27.6)	<0.001	<0.001^a^
Total RAI of anti-PT IgG (AU), mean (SD)	140.1 (31.9)	117.8 (35.7)	0.012	0.024^b^
Total absolute avidity levels of anti-PT IgG (AAU/mL), GMC (95% CI)	84.9 (69.2–104.1)	24.0 (17.0–33.8)	<0.001	<0.001^a^
**Fractional absolute anti-PT IgG levels (IU/mL) with different avidities, GMC (95% CI)**				
Very low	1.0 (0.6–1.7)	1.4 (0.7–2.5)	0.495	0.629^c^
Low	2.0 (1.3–3.1)	1.3 (0.8–2.2)	0.209	0.672^d^
Low–medium	8.7 (6.8–11.1)	3.0 (1.8–5.1)	0.001	0.005^a^
Medium	8.7 (7.2–10.6)	3.4 (2.2–5.4)	<0.001	<0.001^d^
Medium–high	9.1 (7.2–11.7)	2.8 (1.6–4.8)	<0.001	<0.001^e^
High	7.5 (5.6–9.9)	1.6 (0.9–2.9)	<0.001	<0.001^f^
Very high	9.2 (6.8–12.3)	1.4 (0.8–2.6)	<0.001	<0.001^g^

*PT, pertussis toxin; IgG, immunoglobulin G; Tdap, tetanus-diphtheria and acellular pertussis; RAI, relative avidity index; IU, international unit; SD, standard deviation; AU, Avidity Unit; AAU/mL, Absolute Avidity Unit/mL*.

* *For each specific anti-PT IgG levels, Univariate linear regression analysis was used to identify baseline characteristics variables that could potentially impact the anti-PT IgG levels. This was followed by multi-variate regression model, for each anti-PT IgG levels, that adjusted for potential confounders detected in univariate regression analysis as well vaccination status in pregnancy. The adjusted P-value is presented*.

a *Adjusted for gestational age at birth (weeks), parity (yes, no) and delivery mode (Caesarian Section or vaginal delivery)*.

b *Adjusted for gestation at delivery (≤ 37, >37 weeks) and delivery mode (Caesarian Section or vaginal delivery)*.

c *Adjusted for ethnicity and delivery mode (Caesarian Section or vaginal delivery)*.

d *Adjusted for gestational age at birth (weeks) and gestation at delivery (≤ 37, >37 weeks)*.

e *Adjusted for gestational age at birth (weeks), gestation at delivery (≤ 37, >37 weeks), parity (yes, no) and ethnicity*.

f *Adjusted for gestational age at birth (weeks), parity (yes, no), ethnicity and delivery mode (Caesarian Section or vaginal delivery)*.

g *Adjusted for maternal age (years), gestational age at birth (weeks), gestation at delivery (≤ 37, >37 weeks), parity (yes, no), ethnicity, and delivery mode (Caesarian Section or vaginal delivery)*.

### Anti-PT IgG Avidity by Timing of Vaccination in Pregnancy

**Figure 1:**
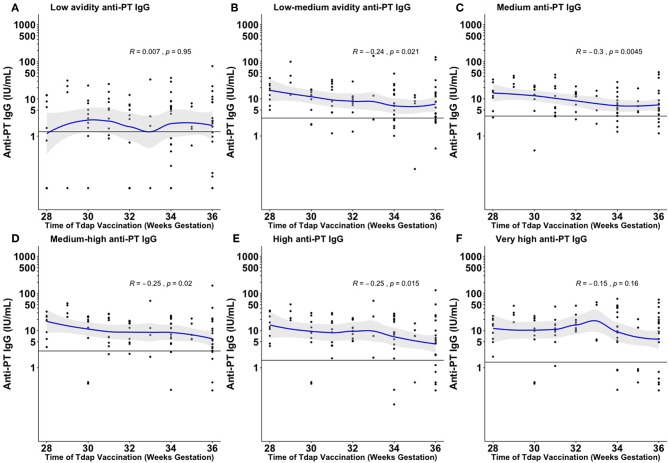
Fractional absolute anti-PT IgG levels by time of vaccination against pertussis in pregnancy achieved at the different ammonium thiocyanate concentrations. The quantified fractional absolute avidity levels of anti-PT IgG at 0.25 molar (M), 0.5, 1, 1.5, 2, and 3 M of ammonium thiocyanate are classified as low **(A)**, low-medium **(B)**, medium **(C)**, medium-high **(D)**, high **(E)**, and very high **(F)** avidity anti-PT IgG antibodies, respectively. The horizontal line denotes the cord mean levels in newborns born to unvaccinated women. PT, pertussis toxin; IU/ml, international unit/ml. This figure shows that the earlier Tdap is given in pregnancy the higher fractional absolute levels of low-medium, medium, medium–high, and high avidity anti-PT IgG levels are achieved at birth.

There was a significant negative association between later timing of Tdap administration and total anti-PT IgG levels and fractional absolute levels of low–medium, medium, medium–high, and high avidity anti-PT IgG (Figure 1 and Supplementary Figure 2).

Newborns of women immunized with Tdap during 28–32 WG had higher total anti-PT IgG levels and fractional absolute levels of medium and high avidity anti-PT IgG compared with newborns of women immunized during 33–36 WG (Table 2). In multivariate analysis, early vaccination remained significantly associated with higher total anti-PT IgG levels and fractional absolute levels of medium and high avidity anti-PT IgG (Table 2).

**Table 2:** Cord anti-PT IgG levels with different avidities of women vaccinated with Tdap during early and late third trimester.

	**Vaccinated during 28–32 WG (*n* = 43)**	**Vaccinated during 33–36 WG (*n* = 47)**	***P***	Adjusted P^*^
Total anti-PT IgG levels (IU/mL), GMC (95% CI)	75.3 (61.2–92.9)	52.66 (39.9–69.6)	0.046	0.038^a^
Total RAI of anti-PT IgG (AU), mean (SD)	136.5 (28.4)	143.3 (34.7)	0.313	0.317^**^
Total absolute avidity levels of anti-PT IgG (AAU/mL), GMC (95% CI)	100.0 (78.3–127.8)	73.1 (53.3–100.3)	0.128	0.119^**^
**Fractional absolute anti-PT IgG levels (IU/mL) with different avidities, GMC (95% CI)**				
Very low	1.2(0.5–2.6)	0.9 (0.5–1.7)	0.667	0.675^b^
Low	2.0 (1.1–3.9)	2.0 (1.1–3.6)	0.986	0.759^c^
Low–medium	11.2 (8.5–14.7)	6.9 (4.7–10.2)	0.051	0.054^**^
Medium	11.6 (8.8–15.2)	6.7 (5.2–8.6)	0.005	0.007^d^
Medium–high	11.4 (8.2–15.8)	7.5 (5.2–10.7)	0.088	0.090^**^
High	10.1 (7.4–13.8)	5.7 (3.6–8.9)	0.042	0.035^a^
Very high	11.2 (8.1–15.3)	7.7 (4.7–12.5)	0.210	0.268^e^

*PT, pertussis toxin; IgG, immunoglobulin G; Tdap, tetanus-diphtheria and acellular pertussis; WG, weeks gestation; RAI, relative avidity index; IU, international unit; SD, standard deviation; AU, Avidity Unit; AAU/mL, Absolute Avidity Unit/mL*.

* *For each specific anti-PT IgG levels, Univariate linear regression analysis was used to identify baseline characteristics variables that could potentially impact the specific anti-PT IgG levels. This was followed by multi-variate regression model, for each anti-PT IgG levels, that adjusted for potential confounders detected in univariate regression analysis as well timing of vaccination in pregnancy. The adjusted P-value is presented*.

** *No other variable (other than timing of vaccination) with p ≤ 0.25*.

a *Adjusted for gestational age at birth (weeks)*.

b *Adjusted for ethnicity and delivery mode (Caesarian Section or vaginal delivery)*.

c *Adjusted for gestational age at birth (weeks), gestation at delivery (≤ 37, >37 weeks)*.

d *Adjusted for gestation at delivery (≤ 37, >37 weeks)*.

e *Adjusted for ethnicity*.

### Anti-PT IgG Avidity by Interval Between Vaccination to Delivery

**Figure 2:**
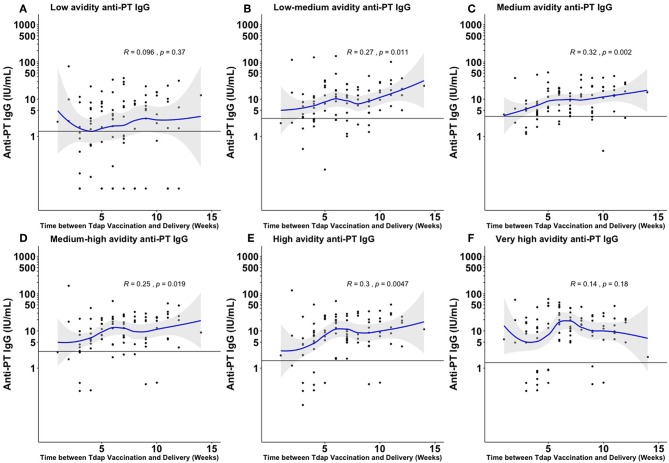
Fractional absolute anti-PT IgG levels by time elapsed from vaccination against pertussis in pregnancy to delivery achieved at the different ammonium thiocyanate concentrations. The quantified fractional absolute avidity levels of anti-PT IgG at 0.25 molar (M), 0.5, 1, 1.5, 2, and 3 M of ammonium thiocyanate are classified as low **(A)**, low-medium **(B)**, medium **(C)**, medium-high **(D)**, high **(E)**, and very high **(F)** avidity anti-PT IgG antibodies, respectively. The horizontal line denotes the cord mean levels in newborns born to unvaccinated women. PT, pertussis toxin; IU/ml, international unit/ml. This figure shows that the longer the interval between Tdap administration during the third trimester and delivery, the higher the fractional absolute levels of low–medium, medium, medium–high, and high avidity anti-PT IgG achieved at birth.

There was a significant positive association between increasing time between Tdap administration during the third trimester and delivery and total anti-PT IgG levels, total absolute avidity levels of anti-PT IgG and fractional absolute levels of low–medium, medium, medium–high, and high avidity anti-PT IgG (Figure 2 and Supplementary Figure 3).

No significant differences were observed in anti-PT IgG of newborns born to women vaccinated 5–8 vs. 9–12 weeks prior to delivery, thus the two groups were combined (data not shown). Newborns of women vaccinated with Tdap 5–12 weeks prior to delivery had higher total anti-PT IgG levels, total absolute avidity levels of anti-PT IgG and fractional absolute levels of low-medium, medium, medium-high, high and very high avidity anti-PT IgG compared with newborns of women vaccinated 4 weeks or less prior to delivery (Table 3). In multivariate analysis, vaccination with Tdap 5–12 weeks prior to delivery remained significantly associated with higher total anti-PT IgG levels, total absolute avidity levels of anti-PT IgG and fractional absolute anti-PT IgG levels of low–medium, medium, medium–high, high and very high avidity anti-PT IgG compared to newborns of women vaccinated 4 weeks or less prior to delivery (Table 3).

**Table 3:** Cord anti-PT IgG levels with different avidities of women vaccinated at different time intervals prior to delivery.

**Time interval between vaccination and delivery**	Vaccinated 1–4 weeks prior to delivery^*^ (*n* = 25)	**Vaccinated 5–12 weeks prior to delivery (*n* = 64)**	***P***	Adjusted P^**^
Total anti-PT IgG levels (IU/mL), GMC (95% CI)	37.2 (25.3–54.8)	76.2 (63.6–91.3)	0.002	<0.001^a^
Total RAI of anti-PT IgG (AU), mean (SD)	132.9 (33.8)	143.6 (30.5)	0.174	0.151^***^
Total absolute avidity levels of anti-PT IgG (AAU/mL), GMC (95% CI)	47.8 (30.9–73.9)	105.9 (86.3–130.0)	0.002	<0.001^***^
**Fractional absolute anti-PT IgG levels (IU/mL) with different avidities, GMC (95% CI)**				
Very low	1.5 (0.7–3.3)	0.9 (0.5–1.6)	0.282	0.378^b^
Low	1.6 (0.8–3.4)	2.2 (1.3–3.6)	0.522	0.950^c^
Low–medium	5.6 (3.5–9.0)	10.2 (7.7–13.5)	0.039	0.030^***^
Medium	5.0 (3.6–7.0)	10.7 (8.6–13.3)	<0.001	<0.001^a^
Medium–high	5.1 (2.9–9.1)	11.5 (8.9–14.6)	0.016	0.005^***^
High	3.3 (1.7–6.4)	10.3 (7.9–13.4)	0.004	<0.001^a^
Very high	4.3 (2.2–8.5)	12.6 (9.4–16.9)	0.007	0.002^d^

*PT, pertussis toxin; IgG, immunoglobulin G; Tdap, tetanus-diphtheria and acellular pertussis; WG, weeks gestation; RAI, relative avidity index; IU, international unit; SD, standard deviation; AU, Avidity Unit; AAU/mL, Absolute Avidity Unit/mL*.

* *One newborn born to woman vaccinated 1 week before delivery, 3 newborns born to women vaccinated 2 weeks before delivery, 9 newborns born to women vaccinated 3 weeks before delivery and 12 newborns born to women vaccinated 4 weeks before delivery*.

** *For each specific anti-PT IgG levels, Univariate linear regression analysis was used to identify baseline characteristics variables that could potentially impact the specific anti-PT IgG levels. This was followed by multi-variate regression model, for each anti-PT IgG levels, that adjusted for potential confounders detected in univariate regression analysis as well timing between vaccination in pregnancy and delivery. The adjusted P-value is presented*.

*** *No other variable (other than timing of vaccination) with p ≤ 0.25*.

a *Adjusted for gestational age at birth (weeks)*.

b *Adjusted for ethnicity and delivery mode*.

c *Adjusted for maternal age (years)*.

d *Adjusted for ethnicity*.

### Distribution of Anti-PT IgG Avidity by Timing of Vaccination

**Figure 3:**
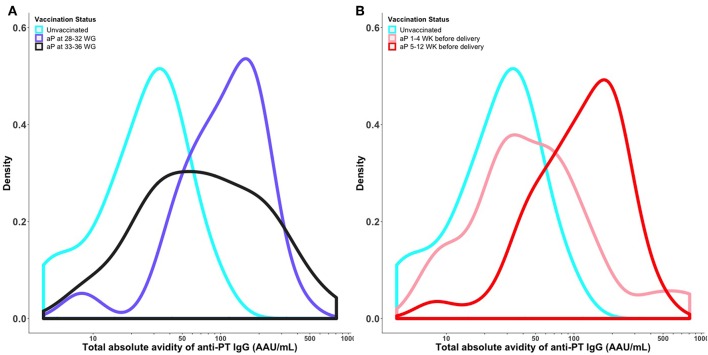
Distribution of total absolute avidity of anti-PT IgG by timing of vaccination in pregnancy **(A)** and time elapsed between vaccination and delivery **(B)**. Kernel Density plot shows the total absolute avidity of anti-PT IgG in cord sera of newborns of women vaccinated against pertussis in pregnancy at different times. The density curves were obtained using a Gaussian kernel. PT, pertussis toxin; aP, acellular pertussis; WG, weeks gestation; WK, weeks; IU/ml, international unit/ml; AAU/mL, Absolute Avidity Unit/mL. This figure shows that vaccination with Tdap during 28–32 WG resulted in higher total absolute avidity levels of anti-PT IgG compared with newborns born to women vaccinated during 33–36 WG. Vaccination with Tdap 5–12 weeks prior to delivery resulted in higher cord total absolute avidity levels of anti-PT IgG compared with vaccination within 4 weeks prior to delivery.

Vaccination with Tdap during 28–32 WG resulted in a shift in the overall distribution of total absolute avidity levels of anti-PT IgG, with higher levels in newborns of women vaccinated during 28–32 WG compared with newborns born to women vaccinated during 33–36 WG. Vaccination with Tdap 5–12 weeks prior to delivery resulted in a shift in the overall distribution of total absolute avidity levels of anti-PT IgG, with higher levels in newborns of women vaccinated 5–12 weeks prior to delivery compared with vaccination within 4 weeks prior to delivery (Figure 3).

### Clustering of Newborns by Anti-PT IgG Avidity

**Figure 4:**
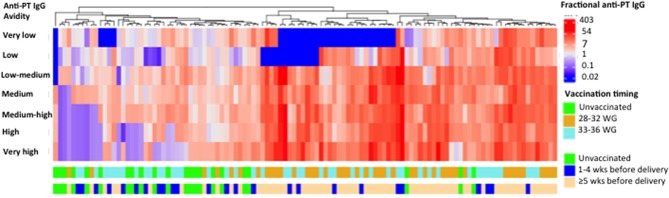
Heat-map analysis based on hierarchical unsupervised clustering. Fractional absolute levels of anti-PT IgG with different avidities for 112 cord samples are illustrated. In the heat-map, natural log fractional absolute anti-PT IgG levels are shown by column. The natural log fractional absolute anti-PT IgG levels were color-coded as indicated by the scale in the right, in which levels range from blue to red indicating high (red) and low (blue) levels. Timing of tetanus diphtheria and acellular pertussis (Tdap) administration is displayed by the different rows. PT, pertussis toxin; IU/ml, international unit/ml; WG, weeks gestation; M, molar; wks, weeks. This figure shows that most newborns of women vaccinated during 28–32 WG or more than (or equal to) 5 weeks prior to delivery had an avidity profile consisting of high levels of high fractional absolute anti-PT IgG levels.

Among newborns of women vaccinated during 28–32 WG, 36/43 (83.7%) had an avidity profile consisting of high levels of high fractional absolute anti-PT IgG levels. Among newborns of women vaccinated more than (or equal to) 5 weeks prior to delivery, 52/65 (80.0%) had an avidity profile consisting of high fractional absolute anti-PT IgG levels (Figure 4).

## Discussion

In this study, we describe a novel approach which enabled comprehensive characterization of the full avidity profile of anti-pertussis antibodies induced by vaccination in pregnancy. This analytical approach can be adopted in studies assessing the avidity of antibodies after vaccination or infection with a range of vaccines and pathogens and settings, beyond pertussis immunization in pregnancy.

We found that newborns of women vaccinated with Tdap in pregnancy had higher total absolute avidity levels of anti-PT IgG, and higher levels of medium to very high avidity anti-PT IgG compared with newborns of unvaccinated women. Furthermore, we found that newborns born to women vaccinated with Tdap during 28–32 WG had higher levels of medium and high avidity anti-PT IgG antibodies compared with newborns born to women vaccinated during 33–36 WG. In addition, newborns of women vaccinated 5–12 weeks prior to delivery achieved higher total absolute avidity levels of anti-PT IgG antibodies, and higher levels of medium to very high avidity anti-PT IgG antibodies compared with vaccination within 4 weeks prior to delivery. This is the first study that characterizes the full avidity profile of anti-PT IgG elicited by pertussis vaccination and highlights important changes in antibody avidity related to timing of vaccination in pregnancy, supporting vaccination in early vs. late third trimester of pregnancy.

Data on the effect of timing of pertussis vaccination in pregnancy on the avidity of anti-pertussis antibodies are scarce. A study by Abu-Raya et al. (35) reported that Tdap vaccination in pregnancy between 27 and 31 WG resulted in higher RAI of cord anti-PT IgG compared with vaccination beyond 31 WG (24). Conversely, in another small study, there was no significant correlation between cord anti-PT IgG RAI and gestational age at vaccination (categorized as <27, 27–30, and 31–36 WG) (25). These two studies used different, fixed, ammonium thiocyanate concentrations, with the former using 0.25 M and the latter using 1.5 M. In these studies, avidity measurement was based on comparison of antibody levels with vs. without addition of a single, fixed concentration of a bond-breaking agent that disrupts binding between antibodies and the target antigen. This arbitrarily separated antibodies into “low” or “high” avidity. Both studies therefore suffer the limitation of reporting results as a single relative measure and thus are only able to provide a limited perspective on antibody avidity. The approach taken in our current study with a range of ammonium thiocyanate concentrations enables complete profiling of avidity of antibodies generated following vaccination. Our large cohort study of pregnant women using in depth profiling of antibody avidity confirms that concentration as well as avidity increase with increasing time elapsed between vaccination and delivery.

The difference in avidity development after antigen exposure is the result of affinity maturation and increased production of antigen-specific antibody-producing plasma cells during maturation of immune response in the germinal center of lymphoid follicles (35–39). Maturation of plasma cells to produce high-affinity antibodies, after an exposure to an antigen, is due to somatic hypermutation in the antibody variable region genes that encode the regions of antibodies that form the interface with the antigens (35). Somatic hypermutation is followed by selection of B cells based on the affinity of their B cell receptors for the specific antigen, with positive selection of B cells with improved affinity for a specific antigen (39). Altogether, avidity maturation in response to an antigen occurs through a process of clonal proliferation, somatic hypermutation, and selection (38). It has been shown that the affinity of antibodies progressively increases over time after antigen exposure (39–43). In addition, the increase in antibody avidity as time after antigen exposure increases has also been shown after vaccination with pertussis vaccine (22, 23, 44), Hib conjugate vaccine (17, 45), and different pneumococcal vaccines (18). However, those studies used a single concentration of bond-breaking agent in order to assess avidity.

Defining the optimal timing for vaccination against pertussis in pregnancy that provides maximal clinical protection to the infant is important and represents a critical gap in current knowledge (9). Furthermore, data to define the preferred timing of vaccination against pertussis within the third trimester are limited and inconclusive (13, 15). Early third trimester vaccination was associated with infants' lower odds ratio (0.43) to have pertussis infection at age <8 weeks compared with vaccination in late third trimester. However, the findings were limited by the wide confidence intervals of the odds ratio (0.02–7.58) (13). In a small number of infants whose mothers received vaccine up to 1 week before delivery, vaccine effectiveness was 43%, with negative lower limit of the CI, limiting a firm conclusion from this study (15). Vaccination earlier in the third trimester also has the added advantage to increase immunization opportunities for pregnant women subsequently giving birth to premature infants. In the UK, among infants <3 months of age with laboratory–confirmed pertussis, 10/66 were premature (born at 32–36 WG) (14). The latter findings, combined with our data that vaccination more than 4 weeks before delivery provides higher levels of high and very high avidity of anti-pertussis antibodies, suggests that vaccination earlier in the third trimester of pregnancy should be preferred. Although the clinical significance of high levels of medium and high avidity anti-PT IgG needs further study, function of antibodies may correlate with protection from infections. Function of anti-pertussis antibodies is important, as there is no anti-pertussis antibody level that correlates with protection. Function of meningococcal vaccine-induced antibodies (bactericidal titers) has been found to correlate with protection from the meningococcal disease (19). In addition to the highest vaccine-induced immune responses, there are different variables that affect the uptake of a vaccine in pregnancy that need to be considered when determining and recommending the ideal timing of pertussis vaccination in pregnancy. The uptake of a specific vaccine in pregnancy is influenced by timing and number of antenatal care visits, timing of administration of other vaccines (e.g., influenza) and access to vaccination services (46). Achieving the highest vaccine-uptake is ideal for optimal protection against pertussis disease in infancy and should be balanced against vaccination in a narrow window that is associated with the highest vaccine-induced immune response.

Our study has a number of strengths. This is the first detailed characterization of full antibody avidity spectrum of anti-pertussis antibodies in a large cohort of well-characterized subjects. The use of a concentration gradient of bond-breaking agent enabled accurate quantification of fractional absolute anti-PT IgG levels according to their binding characteristic to PT antigen. The high linear correlation between anti-PT IgG RAI and ammonium thiocyanate, within the range of ammonium thiocyanate concentration used in the study, enabled the calculation of a weighted measure of total RAI and the quantification of total absolute avidity levels of anti-PT IgG. Altogether, this analytical approach enabled us to perform novel and in-depth analyses of the immune response following vaccination in pregnancy and to link it to clinical variables. The calculated single value of total absolute avidity levels of anti-PT IgG, incorporating both antibody quantity and function (avidity) can be used in future research aimed at establishing correlates of protection against pertussis. In addition, given the ability to characterize the composition of antibodies with different avidity characteristics, we were also able to show that newborns can be clustered according to the timing of vaccination in the third trimester.

This study also has some limitations. The effect of timing during the third trimester was explored; however, optimal timing of vaccination during the entire pregnancy should be explored, as vaccination against pertussis in the second trimester has been shown to be associated with higher anti-pertussis antibody levels as compared to vaccination in the third trimester (47). Our study was not a randomized controlled trial and there were imbalances in some baseline characteristics (e.g., mode of delivery) between the different cohorts; however, adjustments were made for co-variates in multivariable analysis. Including women vaccinated against pertussis within 5 years before pregnancy, and their high percentage in unvaccinated women, is another limitation as it could have affected the results of the comparison of anti-PT IgG levels of newborns born to vaccinated and unvaccinated women. However, the GMC of anti-PT IgG levels of this subgroup was 18.6 IU/mL and thus it is expected to have has minimal effect on anti-PT IgG. A study by Abu Raya et al. followed women vaccinated with Tdap during the third trimester of pregnancy and reported that anti-PT IgG levels declined significantly from 21.5 to 11.7 IU/mL, 9–15 months after delivery (48). A study by Maertens et al. found that after pre-pregnancy Tdap vaccination, anti-PT IgG levels decreased significantly from 69.9 EU/mL 1-month post-vaccination to 13.43 EU/mL at delivery (within a mean interval of 16 months after pre-pregnancy Tdap vaccination) (49). The small number of the study participants vaccinated during early vs late third trimester might have limited the ability to detect significant differences in the anti-PT IgG levels achieved at 0.5 and 1.5 M of ammonium thiocyanate. In addition, our study included only cord sera for analysis and did not include premature infants. Thus, additional studies in preterm infants are needed to investigate the avidity profile of preterm infant born within short time after maternal pertussis vaccination in pregnancy. In our study, only one full-term infant was born 1 week after maternal Tdap vaccination in pregnancy, a time period not sufficient for induction of immune response. Thus, the inclusion of this newborn is unlikely to affect our results. Lastly, our data did not have full details of previous pertussis immunization of the participants.

In conclusion, this is the first study that characterizes in-depth the profile of the avidity of anti-pertussis antibodies elicited by vaccination in pregnancy and it's relation to timing of vaccination in pregnancy. Neonates born to women vaccinated against pertussis during 28–32 WG had higher levels of medium and high avidity anti-pertussis antibodies compared with newborns born to women vaccinated during 33–36 WG. Furthermore, most newborns of women vaccinated during 28–32 WG have avidity profile consisting of high levels of high avidity anti-pertussis antibodies. Future studies need to determine the profile of avidity of anti-pertussis antibodies that is generated after vaccination even earlier in pregnancy and to determine the correlation of these findings with clinical protection from pertussis disease.

## Data Availability Statement

The datasets for this manuscript are not publicly available because of constraints based on the original consent provided by study participants. Requests to access the datasets should be directed to Dr. BA-R.

## Ethics Statement

This original study was carried out in accordance with the recommendations of Monash Health Human Research Ethics Committee (HREC Ref: 13426B) and all participants provided informed and signed consent. The current study was approved by University of British Columbia Children's and Women Research Ethics Board (Certificate number: H17–00050).

## Author Contributions

BA-R conceived and designed the current study, analyzed and interpreted the data, and wrote the first manuscript draft. MG conceived and designed the original and current study, interpreted the data, critically reviewed, and edited the manuscript. TK conceived and designed the current study, interpreted the data, critically reviewed, and edited the manuscript. MS conceived and designed the current study, analyzed and interpreted the data, critically reviewed, and edited the manuscript. All authors reviewed and approved the final version of the manuscript.

### Conflict of Interest

MS is supported via salary awards from the BC Children's Hospital Foundation, the Canadian Child Health Clinician Scientist Program and the Michael Smith Foundation for Health Research. MS has been an investigator on studies funded by Pfizer, Merck, VBI Vaccines and GSK. All funds have been paid to his institute, and he has not received any personal payments. The remaining authors declare that the research was conducted in the absence of any commercial or financial relationships that could be construed as a potential conflict of interest.
