# Supplementary material for: The Effect of Timing of Tetanus-Diphtheria-Acellular Pertussis Vaccine Administration in Pregnancy on the Avidity of Pertussis Antibodies
Source: Front Immunol. 2019 Oct 11;10:2423. doi: 10.3389/fimmu.2019.02423 (PMC6798090; doi:10.3389/fimmu.2019.02423)
Supplement: Supplementary file 1 [file Data_Sheet_1.docx]

**Supplementary Material**

**Supplementary Table 1a:** Calculation of relative avidity index, fractional relative avidity index, total relative avidity index and quantification of fractional and absolute avidity levels of anti-PT IgG.

**Supplementary Table 1b**: Example of calculation of relative avidity index, fractional relative avidity index, total relative avidity index and quantification of fractional and absolute avidity levels of anti-PT IgG.

**Supplementary Figure 1.** Flow chart of study participants

**Supplementary Table 2.** Demographic and baseline characteristics of study participants

**Supplementary Figure 2.** Cord (A) total anti-PT IgG, (B) total relative avidity index, (C) total absolute avidity levels anti-PT IgG levels by time of vaccination against pertussis in pregnancy.

**Supplementary Figure 3.** Cord (A) Total anti-PT IgG, (B) total relative avidity index, (C) total absolute avidity levels anti-PT IgG levels by time elapsed from vaccination against pertussis in pregnancy to delivery.

**Supplementary Table 1a: Calculation of relative avidity index, fractional relative avidity index, total relative avidity index and quantification of fractional and absolute avidity levels of anti-PT IgG.**

| **NH_4_SCN Concentration (molar [M])** | **3 M** | **2 M** | **1.5 M** | **1 M** | **0.5 M** | **0.25 M** | **0 M** | **NA***** |
| --- | --- | --- | --- | --- | --- | --- | --- | --- |
| **Anti-PT IgG levels (IU/mL)** | T_3_ | T_2_ | T_1.5_ | T_1_ | T_0.5_ | T_0.25_ | T_0_ | NA |
| **RAI^*^ (%)** | RAI_3_= T_3_/T_o_*100 | RAI_2_= T_2_/T_o_*100 | RAI_1.5_= T_1.5_/T_o_*100 | RAI_1_= T_1_/T_o_*100 | RAI_0.5_= T_0.5_/T_o_*100 | RAI_0.25_= T_0.25_/T_o_*100 | NA | NA |
| **Fractional (F) RAI (%)** | F RAI_3_= RAI_3_ | F RAI_2_= RAI_2_-RAI_3_ | F RAI_1.5_= RAI_1.5_-RAI_2_ | F RAI_1_= RAI_1_-RAI_1.5_ | F RAI_0.5_= RAI_0.5_-RAI_1_ | F RAI_0.25_= RAI_0.25_-RAI_0.5_ | NA | F RAI_<0.25_= 100% - RAI_0.25_ |
| **Total RAI (AU)** | F RAI_3_*3 + F RAI_2_*2+ F RAI_1.5_*1.5+ F RAI_1_*1+ F RAI_0.5_*0.5+ F RAI_0.25_*0.25+ F RAI_<0.25_*0.125 | | | | | | | |
| **Fractional (F) absolute (abs) avidity levels^**^ (IU/mL)** | F abs_3_=F RAI_3_*T_0_ | F abs _2_=F RAI_2_*T_0_ | F abs_1.5_=F RAI_1.5_*T_0_ | F abs_1_=F RAI_1_*T_0_ | F abs_0.5_=F RAI_0.5_*T_0_ | F abs _0.25_=FRAI_0.25_*T_0_ | NA | F abs _<0.25_=FRAI_0<0.25_*T_0_ |
| **Total absolute avidity levels (AAU/mL)** | F abs_3_*3 + F abs_2_*2+ F abs_1.5_*1.5+ F abs_1_*1+ F abs_0.5_*0.5+ F abs_0.25_*0.25+ F abs_<0.25_*0.125 | | | | | | | |

Abbreviations: PT: pertussis toxin; IgG: immunoglobulin G; M: molar; NA: not applicable; IU:mL: international unit/ml; T: total; RAI: relative avidity index; F: fractional; AU: Avidity Unit; AAU/mL: Absolute Avidity Unit/mL; abs: absolute.

* Samples treated with PBS or the lowest NH_4_SCN concentration (0.25M NH_4_SCN) with optic density values lower than the ELISA’s lower levels of quantification (LLOQ) were excluded from further avidity analysis. Samples treated with 0.5M, 1M, 1.5M, 2M, 3M concentrations of NH_4_SCN and with optic density values lower than the ELISA’s LLOQ were assigned an arbitrary RAI value of 2.5%, 5%, 7.5%, 10% and 12.5%, respectively, for the respective NH_4_SCN concentrations.

** Fractional absolute avidity levels of anti-PT IgG at a specific NH_4_SCN concentration quantified as 0 were assigned an arbitrary value of 0.04 IU/mL

*** This column includes the Fractional (F) RAI and Fractional (F) absolute (abs) avidity levels of anti-PT IgG antibodies eluted at the lowest NH_4_SCN concentration

**Supplementary Table 1b: Example of calculation of relative avidity index, fractional relative avidity index, total relative avidity index and quantification of fractional and absolute avidity levels of anti-PT IgG.**

| **NH_4_SCN Concentration (molar [M])** | **3 M** | **2 M** | **1.5 M** | **1 M** | **0.5 M** | **0.25 M** | **0 M** | **NA***** |
| --- | --- | --- | --- | --- | --- | --- | --- | --- |
| **Anti-PT IgG levels (IU/mL)** | 18 IU/mL | 42 IU/mL | 60 IU/mL | 84 IU/mL | 96 IU/mL | 108 IU/mL | 120 IU/mL | NA |
| **RAI^*^ (%)** | 18/120*100=15% | 42/120*100=35% | 60/120*100=50% | 84/120*100=70% | 96/120*100=80% | 108/120*100=90% | NA | NA |
| **Fractional (F) RAI (%)** | 15% | 35%-15%=20% | 50%-35%=15% | 70%-50%=20% | 80%-70%=10% | 90%-80%=10% | NA | 100%-90%=10% |
| **Total RAI (AU)** | 15%*3+ 20%*2 + 15%*1.5 + 20%*1 + 10%*0.5 + 10%*0.25 + 10%*0.125= 136.25 AU | | | | | | | |
| **Fractional (F) absolute (abs) avidity levels^**^ (IU/mL)** | 15%*120=18 IU/mL | 20%*120= 24 IU/mL | 15%*120= 18 IU/mL | 20%*120=24 IU/mL | 10%*120=12 IU/mL | 10%*120= 12 IU/mL | NA | 10%*120= 12 IU/mL |
| **Total absolute avidity levels (AAU/mL)** | 18*3 + 24*2 + 18*1.5 + 24*1 + 12*0.5 + 12*0.25 + 12*0.125= 163.5 AAU/mL | | | | | | | |

Abbreviations: PT: pertussis toxin; IgG: immunoglobulin G; M: molar; NA: not applicable; IU:mL: international unit/ml; T: total; RAI: relative avidity index; F: fractional; AU: Avidity Unit; AAU/mL: Absolute Avidity Unit/mL; abs: absolute.

* Samples treated with PBS or the lowest NH_4_SCN concentration (0.25M NH_4_SCN) with optic density values lower than the ELISA’s lower levels of quantification (LLOQ) were excluded from further avidity analysis. Samples treated with 0.5M, 1M, 1.5M, 2M, 3M concentrations of NH_4_SCN and with optic density values lower than the ELISA’s LLOQ were assigned an arbitrary RAI value of 2.5%, 5%, 7.5%, 10% and 12.5%, respectively, for the respective NH_4_SCN concentrations.

** Fractional absolute avidity levels of anti-PT IgG at a specific NH_4_SCN concentration quantified as 0 were assigned an arbitrary value of 0.04 IU/mL

*** This column includes the Fractional (F) RAI and Fractional (F) absolute (abs) avidity levels of anti-PT IgG antibodies eluted at the lowest NH_4_SCN concentration

**Supplementary Figure 1: Flow chart of study participants**


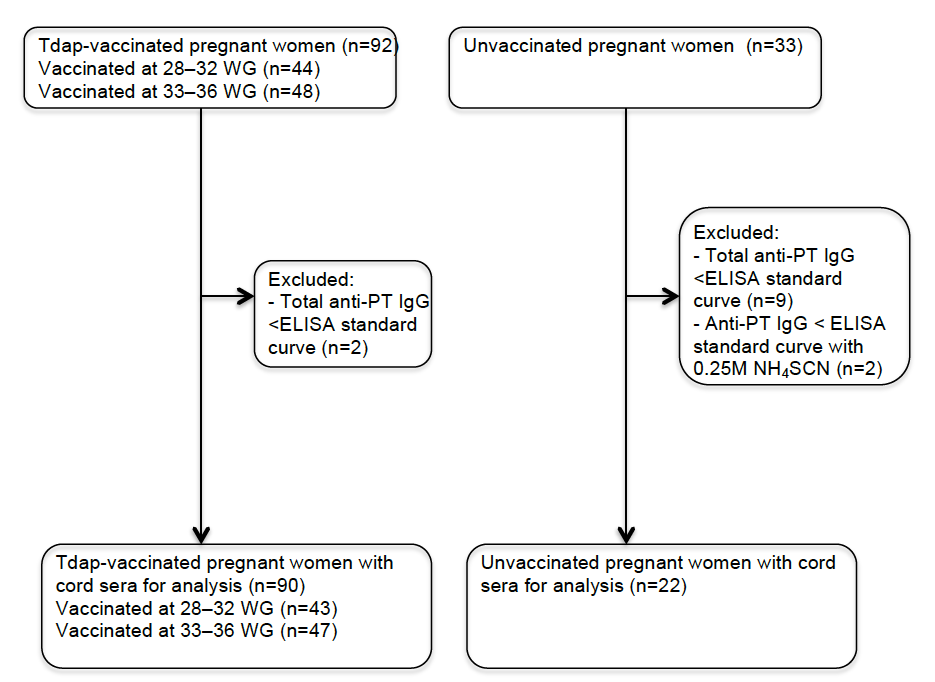


Abbreviations: Tdap: tetanus-diphtheria acellular pertussis; WG: weeks gestation; PT: Pertussis toxin, IgG: immunoglobulin G, ELISA: enzyme-linked immunosorbent assay.

**Supplementary Table 2: Demographic and baseline characteristics of study participants**

| Characteristic | Tdap-vaccinated (n=90) | Unvaccinated (n=22) | P^1^ | Vaccinated during 28–32 WG  (n=43) | Vaccinated during  33–36 WG  (n=47) | P^2^ | P^3^ |
| --- | --- | --- | --- | --- | --- | --- | --- |
| **Ethnicity** | | | | | | |  |
| Australian born, n (%) | 44 (48.9) | 11 (50) | 1 | 23 (53.5) | 21 (44.7) | 0.533 | 0.703 |
| **Maternal age, years** | | | | | | |  |
| Mean (SD) | 30.0 (4.4) | 32.1 (4.8) | 0.072 | 30.3 (4.2) | 29.8 (4.6) | 0.531 | 0.126 |
| **Parity** | | | | | | |  |
| Nulliparous, n (%) | 46 (51.1) | 5 (22.7) | **0.031** | 21 (48.8) | 25 (53.2) | 0.840 | 0.052 |
| **Mode of delivery, n (%)** | | | | | | |  |
| Elective CS | 6 (6.7) | 15 (68.2) | **<0.001** | 3 (6.9) | 3 (6.4) | 0.882 | **<0.001** |
| Emergency CS | 15 (16. 7) | 0 (0) |  | 8 (18.6) | 7 (14.9) |  |  |
| NVD | 50 (55.5) | 5 (22.7) |  | 22 (51.1) | 28 (59.6) |  |  |
| Instrumental | 19 (21.1) | 2 (9.1) |  | 10 (23.2) | 9 (19.1) |  |  |
| **Gestational age at delivery, weeks** | | | | | | |  |
| Mean (SD) | 39.3 (1.3) | 38.6 (1.1) | **0.015** | 39.2 (1.4) | 39.4 (1.2) | 0.523 | 0.054 |
| **Gestational age at delivery, n (%)** | | | | | | |  |
| <37 weeks | 1^4^ (1.1) | 1^4^ (4.5) | 0.386 | 1 (2.3) | 0 (0) | 0.454 | 0.529 |
| 37–42 weeks | 86 (95.6) | 21 (95.5) |  | 40 (93.0) | 46 (97.9) |  |  |
| >42 weeks | 3 (3.3) | 0 (0) |  | 2 (4.6) | 1 (2.1) |  |  |
| **Pertussis vaccination history before pregnancy** | | | | | | |  |
| No vaccination | 8 (8. 9) | 2 (9. 1) | **<0.001** | 5 (11.6) | 3 (6.4) | 0.616 | **0.001** |
| Not sure | 49 (54.4) | 5 (22.7) |  | 23 (53.5) | 26 (55.3) |  |  |
| Less than 5 years before pregnancy | 7 (7. 8) | 10 (45.4) |  | 2 (4.6) | 5 (10.6) |  |  |
| More than 5 years before pregnancy | 26 (28. 9) | 5 (22.7) |  | 13 (30.2) | 13 (27.6) |  |  |
| Gestational age at vaccination (weeks gestation) | | | | | | | |
| Mean (SD) | 32.6 (2.7) | NA |  | 30.2 (1.4) | 34.9 (1.1) | <0.001 | NA |
| **Time between vaccination and delivery** | | | | | | |  |
| Time interval in weeks (SD) | 6.7 (2.9) | NA |  | 9.0 (2.0) | 4.5 (1.5) | **< 0.001** | **NA** |

Abbreviations: Tdap: tetanus-diphtheria and acellular pertussis; WG: weeks gestation; SD: standard deviation; CS: caesarean section; NVD: normal vaginal delivery; NA: not applicable.

**^1^ P-value for comparison between women vaccinated with Tdap vs. unvaccinated**

**^2^ P-value for comparison between women vaccinated with Tdap during 28-32 WG vs. 33-36 WG**

**^3^ P-value for comparison between women vaccinated with Tdap during 28-32 WG vs 33-36 WG**

**^4^ Born at 36 WG**

**
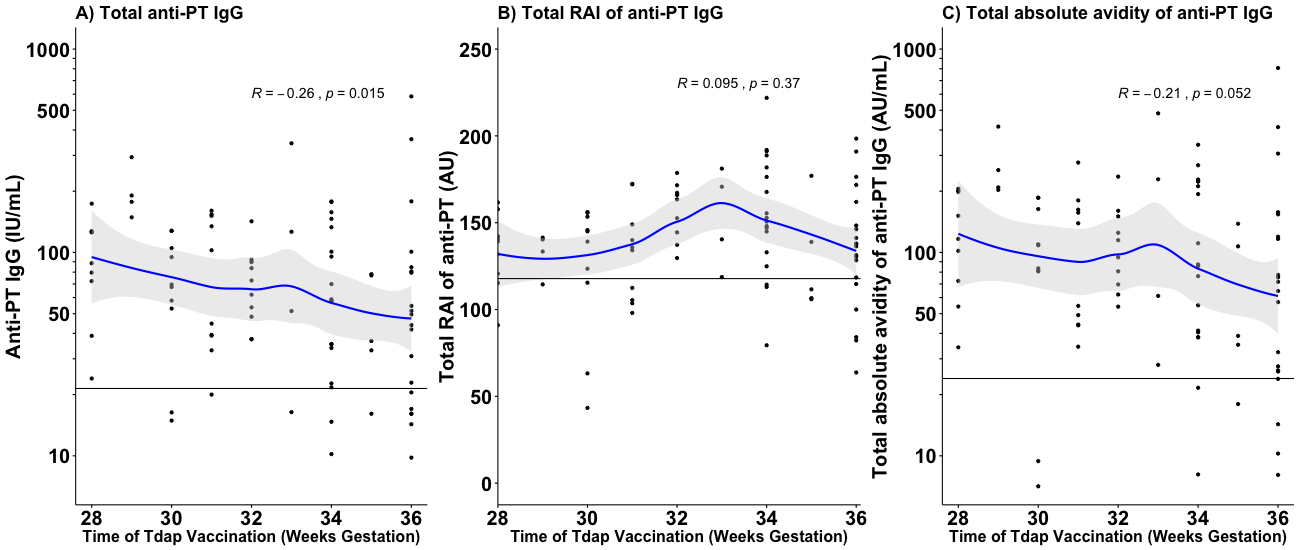
**

**Supplementary Figure 2:** Cord (A) total anti-PT IgG, (B) total relative avidity index, (C) total absolute avidity levels anti-PT IgG levels by time of vaccination against pertussis in pregnancy. The horizontal line denotes the cord mean levels in newborns born to unvaccinated women. Abbreviations: PT: pertussis toxin; RAI: relative avidity index; IU/ml: international unit/ml; AU: Avidity Unit; AAU/mL: Absolute Avidity Unit/mL. This figure shows that the earlier Tdap is given in pregnancy the higher total anti–PT levels are achieved at birth.

**
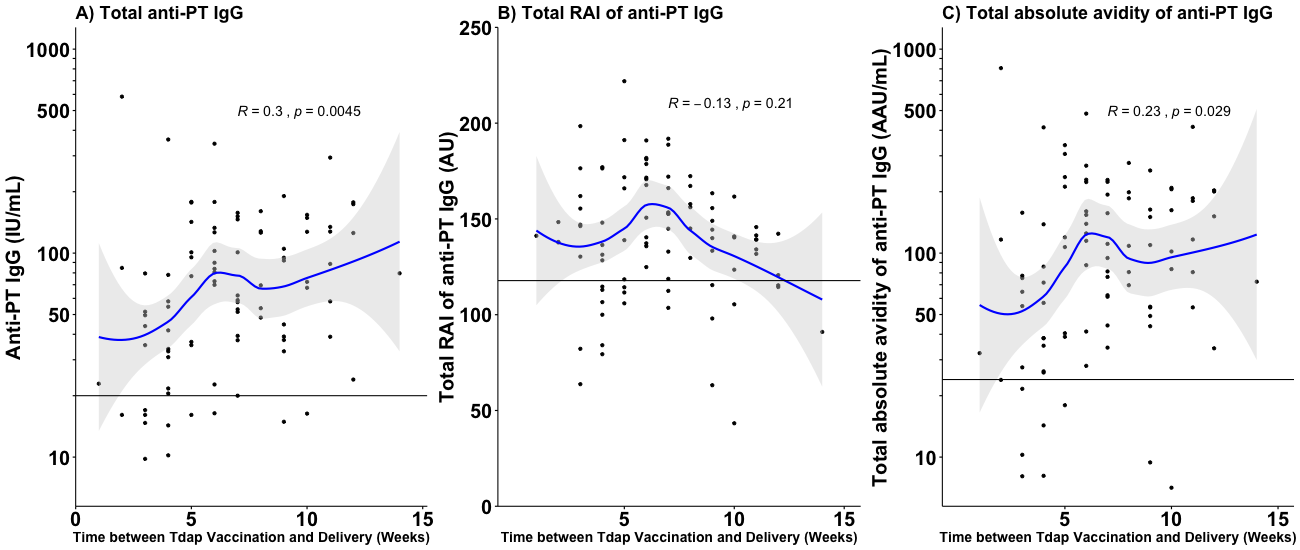
**

**Supplementary Figure 3:** Cord (A) Total anti-PT IgG, (B) total relative avidity index, (C) total absolute avidity levels anti-PT IgG levels by time elapsed from vaccination against pertussis in pregnancy to delivery. The horizontal line denotes the cord mean levels in newborns born to unvaccinated women. Abbreviations: PT: pertussis toxin; RAI: relative avidity index; IU/ml: international unit/ml; AU: Avidity Unit; AAU/mL: Absolute Avidity Unit/mL. This figure shows that the longer the interval between Tdap administration during the third trimester and delivery, the higher the total anti–PT IgG levels and total absolute avidity levels of anti–PT IgG achieved at birth.
